# Perceived Homonegativity and Psychological Distress in Gay Men in Brazil: Does Skin Color Matter?

**DOI:** 10.3390/healthcare13091030

**Published:** 2025-04-30

**Authors:** Felipe Alckmin-Carvalho, Renata Della Torre, Iara Teixeira, Jóni Ledo, António Oliveira, Lúcia Yasuko Izumi Nichiata, Henrique Pereira

**Affiliations:** 1 Department of Psychology and Education, Faculty of Social and Human Sciences, University of Beira Interior, Pólo IV, 6200-209 Covilhã, Portugal; renata.torre@ubi.pt (R.D.T.); iara.teixeira@ubi.pt (I.T.); joni.ledo@ubi.pt (J.L.); antonio.oliveira@ubi.pt (A.O.); hpereira@ubi.pt (H.P.); 2 School of Nursing, University of São Paulo, São Paulo 01239-020, Brazil; izumi@usp.br; 3 School of Psychology, University of Minho, Gualtar Campus, 4710-057 Braga, Portugal; 4 Research Centre in Sports Sciences, Health Sciences and Human Development (CIDESD), 5001-801 Vila Real, Portugal; 5 RISE-Health, Department of Psychology and Education, Faculty of Social and Human Sciences, University of Beira Interior, Pólo IV, 6200-209 Covilhã, Portugal

**Keywords:** male homosexuality, homonegativity, stigma, depression, sexual stigma, racism, LGBTQIA+, mental health

## Abstract

Homonegativity is associated with several adverse physical and mental health outcomes in gay men. However, the intersection between homonegativity and racism remains little investigated in Brazil. The aim of this study was to assess, in a sample of cisgender Brazilian gay men, associations between skin color, homonegativity, psychological distress, and socioeconomic variables. **Method**: A cohort of 229 Brazilian gay men, 151 (66%) white and 78 (34%) black or brown were assessed. Participants completed the Internalized Homophobia Scale, the Beck Depression Inventory-II, and the State–Trait Anxiety Inventory. **Results**: In the general sample, high levels of homonegativity, symptoms of depression, and trait and state anxiety were found, particularly among young and low-income individuals. Black and brown participants reported significantly higher levels of trait and state anxiety, but not depression or homonegativity. Black and brown skin color were a predictor of trait and state anxiety, but not of homonegativity or depression. **Conclusions:** The high levels of psychological distress and homonegativity found in the general sample indicate the importance of developing preventive interventions of racial discrimination and sexual prejudice for the general community and psychoeducational and therapeutic interventions for Brazilian gay men, regardless of skin color. They also suggest the relevance of customizing these interventions to meet the specificities of black/brown Brazilian gay men, a population doubly exposed to the burden of stigma, considering the intersection between racial and sexual stigma in the Brazilian sociocultural reality.

## 1. Introduction

According to the Brazilian Institute of Geography and Statistics [1], 1.8 million Brazilians identify as cisgender gay men. Although Brazil has one of the largest populations of LGBTQIA+ people in the world, and several advances have been made in recent decades regarding the rights of this population, significant challenges remain [2]. In 2024, for example, 225 LGBTQIA+ people were elected during the municipal elections [3], demonstrating progress in the field of political representation in Brazil. However, prejudice persists significantly, as evidenced in various social and political contexts [4].

Brazil is predominantly Christian country, and its social values have historically been influenced by religious discourses portraying homosexuality as sinful or morally deviant [5,6]. In the last few years, Brazil has witnessed a significant increase in fundamentalist evangelical groups, organized in far-right political platforms, with a narrative characterized by intolerance regarding sexual and gender diversity, and racist rhetoric. In 2018, Jair Messias Bolsonaro, later democratically elected as the president of Brazil in 2019, in a speech in the northeastern state of Paraíba, stated, “Minorities must bow down, or they will be crushed” [7], referring to ethnic and sexual minorities. Even in his position as President of the Republic, the politician made many homophobic and racist public speeches, which created a climate of permissiveness among the population [7,8]. Studies indicate that there has been an increase in violence against sexual minorities in Brazil since 2019, with nearly 34,000 complaints of violations of the rights of LGBTQIA+ people registered in the first half of 2024 alone. Of these, 12,000 were specific reports of rights violations involving gay men [9], highlighting the seriousness and scale of the challenges faced by this population.

Brazil is a racially diverse country, made up mainly of brown (45.3%), white (43.5%), black (10.2%), indigenous (0.8%), and East Asian (0.4%) people [10]. However, it is important to note that although black and brown people make up the numerical majority in Brazil, there is still a structural social inequality, originating during European colonization and the late abolition of slavery, which contributes to the social and economic injustice in most of this population [11,12]. In the country, racism manifests itself in subtly different ways in different regions. For example, the Northeast Region is more racially mixed and therefore racial relations tend to be more horizontal, while in the South and Southeast, where racial mixing is less prevalent, racial relations tend to be more vertical [13]. Studies highlight that black Brazilian gay men, in addition to the issues related to homonegativity, are more likely to experience socioeconomic vulnerability, as evidenced by the difficulty in accessing quality education, health, and other basic rights; greater difficulty in finding well-paying jobs and career advancement; religious intolerance; and the lack of representation in positions of power [11,12,13,14]. In addition, young black and brown men in particular are more often victims of more violent police actions because of the color of their skin [12]. Structural racism in Brazil is also evident in an analysis of the profile of incarcerated persons, which is made up of 63% brown and black people, 44% of whom have not completed primary school, almost 30% of whom are still awaiting trial [15] and who have a lower life expectancy than white men, with mortality between the ages of 15 and 34 being particularly driven by “external factors”, which in practice means death by homicide [16].

To avoid disapproval and losing the support of their families and the black community, which are especially important given the broader context of social inequality, many black gay men may choose to keep their sexual identities private, which is associated with poorer quality of life and sexual health indicators [17]. This highly adverse social context increases the chances of engaging in maladaptive coping strategies, such as substance abuse or compulsive sexual behavior. These attempts to alleviate psychological distress and low self-steam related to negative beliefs about their LGBTQIA+ identity and skin color aggravate the repercussions on their mental health [18,19,20].

Experiences of racial discrimination and homonegativity have been widely associated with mental health problems [18,21]. Minority Stress Theory [22] is a fundamental framework for understanding the LGBTQIA+ community [23,24], as it represents individuals who differ from heteronormative norms and are subject to conflicts with a dominant culture that often does not reflect them or and marginalizes their identities through various processes, which culminate in the internalization of stigma by the individual, which may result in internal conflict and psychological distress, as well as increased risk of psychopathologies [22].

In this study, we use the concept of “*homonegativity*”, defined as the expression of negative emotions, thoughts, and behaviors towards LGBTQIA+ individuals [11,25,26,27]. This concept encompasses two main forms: traditional or “*old-fashioned*” homonegativity and “modern homonegativity” [25]. The former is characterized by moral objections, often associated with beliefs in the deterioration of morality or religious principles, while the latter reflects beliefs that LGBTQIA+ people would make demands considered illegitimate to change the social status quo and microaggression [25].

Several studies have demonstrated associations between experiences of homonegativity and negative psychological impacts, involving depression, anxiety, and anger, among other harmful emotional states [4,11,28,29,30,31,32,33]. In this study, both internalized homonegativity and external homonegativity (experienced homonegativity in the community) [34] will be considered, because they are interdependent [35]. When the individual incorporates, often unconsciously, the stigmatizing attitudes imposed by society, they may develop negative attitudes towards themselves and a higher risk of health problems [36]. This phenomenon is known as the “stigma internalization process” [11]. Malyon (1982) [37] postulates that internalized homonegativity is a system of attitudes that has a significant influence on self-concept, self-esteem, and self-care repertoires. This author suggests that the internalization process can occur even before individuals identify themselves as homosexuals [28]. This process encompasses multiple social aspects, including religion, interpersonal relationships, community involvement, and career path, as well as health dimensions such as mental, physical, and sexual health and behavioral aspects [38]. Meyer’s (1995) [28] studies indicate that men who experience homonegativity throughout their development have higher than average levels of internalized homonegativity [5]. As a result, several studies have shown that internalized homonegativity is a predictor of psychological distress [28,38,39,40], which includes symptoms of depression [5,36,41], anxiety [38,41], and shame [21], as well as expectations of rejection, discrimination and events involving violence [28], interpersonal problems, and loneliness [41].

This study also adopts the perspective of Intersectionality Theory, as it seeks to understand the unique experiences of individuals who face multiple forms of inequality and social discrimination [42]. Studies suggest that black gay men face multiple derogatory stereotypes that overlap with stereotypes associated solely with homosexuality or race [18,43,44,45,46,47]. Moreover, the study by Calabrese et al. (2018) [47] indicates that these adverse experiences may occur even within the LGBTQIA+ and black community. These findings suggest a possible lack of social support and sense of belonging even within socially marginalized communities, which increases the risk of negative physical and mental health outcomes [47,48,49,50].

Although people, both heterosexual and LGBTQIA+, believe that policies protecting minorities have already been successfully implemented in the last few years, recent studies show that homonegativity and discrimination continue to exist in the 21st century [29]. For example, a study of three generations, born between 1956 and 1997, found that despite legal advances protecting members of the LGBTQIA+ community, younger people did not present lower levels of psychological distress or reduction in suicide attempts compared to older people [51]. Despite recent advances in rights, LGBTQIA+ people are still oppressed in many countries [52]. In this context, It is imperative that more studies be conducted, particularly focusing on people on the margins of sexual and racial intersectionality, who are often even more marginalized. Research on these populations is essential to better understand the complex dynamics of oppression that these people face and to promote more inclusive and equitable representation in society. Although Minority Stress Theory and Intersectionality Theory postulate that LGBTQIA+ individuals who also belong to and ethnic/racial minority experience higher levels of homonegativity and psychological distress because they conflict with the dominant social structure in two different ways [22,28,33,38,46,53,54], there is no consensus on these findings in the current literature [17,49,50,55].

Thus, to fill a gap on this topic, the general aim in this study was to investigate, in a sample of cisgender Brazilian gay men, associations between skin color, homonegativity, psychological distress, and socioeconomic variables. Specific objectives were to evaluate (a) associations between sociodemographic variables, homonormativity, psychological distress, and skin color; (b) possible differences in homonormativity and psychological distress between black/brown and white gay men; and (c) the predictive power of skin color in homonormativity and in symptoms of depression and anxiety.

To the best of our knowledge, this is the first study that has attempted to assess the level of internalized homonegativity and perceived homonegativity in community among Brazilian gay men considering skin color as a variable of interest. The hypotheses, developed from the perspective that black/brown gay men are doubly exposed to the burden of social stigma, are as follows: H1: there is a significant positive correlation between perceived homonegativity, in community and internalized, and psychological distress; H2: black/brown gay men report more experiences related to homonegativity and refer to more symptoms of depression and anxiety compared to white gay men; and H3: black or brown skin color is a positive predictor of higher scores of homonegativity and symptoms of depression and anxiety.

## 2. Materials and Methods

This is a cross-sectional and comparative study. The sample consisted of 229 self-identified Brazilian gay men, with a mean age of 35.5 years (SD = 8.83). This was a non-probabilistic sample, selected based on convenience criteria. Participants were recruited on social media such as Instagram and Facebook. The inclusion criteria were as follows: self-declaring as cisgender and gay, being 18 years old or older, and having access to the internet and the possibility of filling out the assessment instruments in privacy. To compose the groups of white and black/brown gay men, a question from the sociodemographic questionnaire about skin color was used, with the following possible answers: white, brown (mestizo), East Asian or other. The implications of self-declaration of skin color based on previously established categories, as well as the inclusion of black and brown individuals in the same group, are presented in this article’s Discussion Session.

### 2.1. Instruments

#### 2.1.1. Internalized Homophobia Scale

It is important to note that the name of the instrument we used is the “Internalized Homophobia Scale”. However, we do not consider the term “homophobia” to be the most appropriate to describe the phenomenon we are investigating. We prefer the term “homonegativity”, which is broader and encompasses less obvious forms of discrimination and prejudice, including microaggressions and modern homonegativity. Although the name of the instrument is “Internalized Homophobia Scale”, the scale assesses both internalized homonegativity and perceived homonegativity in the community. The Internalized Homophobia Scale, developed by Ross and Rosser [56], evaluates two dimensions: internalized homonegativity and perceived homonegativity in the community. All items are written in the affirmative form, and measurement is carried out through a 5-point Likert scale ranging from 1 (strongly disagree) to 5 (strongly agree). Examples of the statements are as follows: (1) “Typically, effeminate gay men make me feel uncomfortable”; (2) “I prefer to have anonymous sexual partners”; and (3) “Life would be easier if I were heterosexual”. Higher scores indicate higher levels of internalized homophobia. No cut-off point exists for the classification of homonegativity. In the present study, we used the 19-item Brazilian version of the scale, which showed better internal validity in a validation article [34], with a Cronbach’s alpha of 0.814 for the internalized homonegativity and 0.622 for perceived homonegativity in the community. For the data collected in our study, internalized homophobia had a Cronbach’s alpha of 0.81, while external homophobia had a Cronbach’s alpha of 0.63. When we divide the sample between skin color groups, internal homonegativity reaches 0.80 for the black/brown group and 0.82 for the white group. External homonegativity obtained an alpha of 0.61 for black and brown participants and 0.63 for white participants.

#### 2.1.2. Beck Depression Inventory (BDI-II)

The BDI-II is a widely used instrument that comprises 21 items, each with four alternatives related to the presence and severity of depression symptoms, ranging from 0 to 3 [57]. The questions encompass physical symptoms, such as fatigue, sleep, and weight changes, and cognitive changes verified in patients diagnosed with depression, such as persistent sadness, pessimism, feelings of failure, dissatisfaction, and guilt. The level of depression was classified according to the total score: 0–11 = minimal; 12–19 = mild; 20–35 = moderate; and 36–63 = severe. In a validation study of the instrument in a Brazilian population, a Cronbach’s alpha of 0.81 was obtained [58].

#### 2.1.3. State–Trait Anxiety Inventory (STAI)

The STAI was developed to assess anxiety in two distinct dimensions: (1) trait anxiety, which assesses an individual’s general anxiety level over time, and (2) state anxiety, which evaluates how the individual feels at the current moment [59]. Both scales consist of 20 items related to anxiety symptoms. Example items include the following: (1) “I worry too much over something that really doesn’t matter”, and (2) “I feel nervous”. Responses are rated on a 4-point Likert scale (1 = not at all to 4 = very much), with higher scores indicating greater anxiety. The total scores range from 20 to 80 for both state and trait anxiety, with no specific cutoff for anxiety classification. In the validation study for the Brazilian population, the instrument demonstrated good reliability, with Cronbach’s alphas ranging from 0.86 to 0.95 for state anxiety and from 0.89 to 0.92 for trait anxiety [60]. In the current study, the STAI showed acceptable reliability, with a Cronbach’s alpha of 0.91.

Sociodemographic Questionnaire: developed to assess age, housing condition, employment status, marital status, living arrangements, education level, occupation, HIV-status, religion, and income.

### 2.2. Procedures and Ethical Considerations

Participants in this study responded anonymously and confidentially to the instruments provided on an online platform between January and June 2023. The average time spent completing the instruments was approximately 30 min. This study was promoted through social media, and participants were selected from Facebook groups directed at the LGBTQIA+ community. The research protocol was periodically published over a three-month period. In the online psychological assessment protocol developed by the first author for this study, inclusion criteria were assessed on the first introductory page. If the participant met the inclusion criteria, they were invited to participate. After reading and agreeing to the consent form, participants had access to the sociodemographic questionnaire, the Internalized Homophobia Scale, the BDI-II, and the STAI. This study was approved by the Research Ethics Committee of the School of Nursing at the University of São Paulo under number 4.601.952, CAAE: 31527820.7.0000.5392, on 19 March 2021. All participants provided written informed consent.

### 2.3. Data Analysis

Statistical analyses were performed using SPSS 29.0, and the significance level was set at 5% (*p* < 0.05). Descriptive analyses were presented as frequencies, proportions, means, medians, and standard deviations. Data normality was assessed using the Shapiro–Wilk tests, and the homogeneity of variances was assessed using Levene’s test. Due to normal distribution, Pearson correlations were conducted to examine relationships between variables. Further analyses included Student’s *t*-test for group comparisons, with effect sizes calculated using Cohen’s d. Furthermore, hierarchical multiple linear regression analyses were conducted to assess whether skin color remained a significant predictor of psychological distress, and internalized homonegativity after controlling for education and income, the sociodemographic variables that showed significant correlations with key outcomes. For each dependent variable—depression, state and trait anxiety, total homonegativity, internalized stigma, and social oppression—skin color was inserted in the first step, followed by education and income in the subsequent steps. This approach allowed us to estimate the unique contribution of each predictor while controlling for potential confounding variables.

The following intervals were adopted to classify the intensity of the correlation between the variables analyzed: from 0 to 0.30, slight correlation; from 0.30 to 0.70, moderate correlation; and from 0.7 to 1, strong correlation between variables [61].

## 3. Results

A total of 229 gay men took part in this study, aged between 19 and 69 years old, with a mean age of 35.3 (SD = 8.8). The mean monthly income was *EUR* 1609 (SD = 1647) with salaries ranging from *EUR* 109 to *EUR* 8182 per month, although most participants (67%) had a monthly income of less than *EUR* 1500. When calculating the average salary, participants who did not answer and participants whose monthly income was less than *EUR* 100 or greater than *EUR* 8182 were excluded, as they were considered outliers. Extremely low values may reflect typing errors or unrepresentative sporadic incomes, while very high values may distort the analysis, considering that Brazil’s average income is significantly lower. Even with the exclusion of outliers, the standard deviation is quite high due to the wide range of monthly incomes. Of the 229 participants, 151 (65.9%) identified as white and 78 (34.1%) as black or brown. Most participants (63.4%) had completed higher education, were employed (87.3%), were single (75.1%) and lived with family or a partner (54.6%). Most participants (62%) lived in the city of São Paulo, the most populous urban conglomerate in Brazil. Of the 218 participants who responded to the HIV testing question, 117 (53.7%) had been tested in the last 6 months. In terms of diagnosis, 36 participants (15.7%) reported being HIV-positive, as can be seen in Table 1.

In addition, we analyzed whether there were significant differences in sociodemographic data according to skin color. The results of the chi-square test revealed statistically significant differences in the variables of employment status, socioeconomic status, marital status and religion. The effect sizes calculated with Cramer’s V for the differences in employment and marital status (V = 0.18–0.19) are small and therefore indicative of differences between groups that are quite modest in their magnitude. In the case of socioeconomic status, (V = 0.23) indicates a small-to-moderate association that is somewhat stronger but still very limited. In comparison to these, regarding religious affiliation, a value of V = 0.30 was found, representing a moderate effect that suggests that within the difference between groups of white and black/brown participants, the variable religious identification has more substantial differentiation.

The descriptive results (Table 2) indicated that the observed means varied in relation to the theoretical means for the variables analyzed. In depression, the mean was 14.03, considerably lower than the theoretical mean of 31.5. Community homonegativity had a mean of 11.41 on a scale of 4 to 16, slightly above the theoretical mean of 10. For internalized homonegativity, the mean was 38.21, close to the theoretical mean of 37.5. The total homonegativity score was 49.63, exceeding the theoretical average of 47.5. State anxiety had a mean of 48.16 and trait anxiety 48.65, both close to the theoretical mean of 50.0. These results indicate that depression and homonegativity levels diverged from expected norms, while anxiety scores remained more in line with theoretical expectations.

We conducted a correlation analysis between the study variables of interest, where depression shows a strong positive correlation with state anxiety (r = 0.75; *p* < 0.001) and trait anxiety (r = 0.80; *p* < 0.001), indicating that higher levels of anxiety are strongly associated with increased depressive symptoms. Furthermore, depression is negatively correlated with education level (r = −0.19; *p* < 0.001), income (r = −0.17; *p* < 0.001), and skin color (r = −0.17; *p* < 0.001), suggesting that higher education and income levels are linked to lower depression, and that individuals with lighter skin tones may report lower levels of depression.

In this study, total homonegativity showed a significant positive correlation with external perception of stigma (r = 0.49; *p* = < 0.01) and internal perception of stigma (r = 0.92; *p* < 0.01). These findings indicate a strong relationship between overall homonegativity and its subcomponents, particularly internal perception of stigma. External perception of stigma is positively correlated with internal perception of stigma (r = 0.13; *p* < 0.05) and trait anxiety (r = 0.13; *p* < 0.05), suggesting that individuals who perceive higher external stigma also tend to internalize stigma more and experience higher levels of trait anxiety. State anxiety is highly correlated with trait anxiety (r = 0.87; *p* < 0.05), confirming the close relationship between these two forms of anxiety. State anxiety is also negatively associated with age (r = −0.15; *p* < 0.05) and income (r = −0.16; *p* < 0.05), and positively with skin color (r = 0.14; *p* < 0.05), indicating that younger individuals, those with lower income, and individuals with darker skin tones experience higher levels of state anxiety.

Trait anxiety, in addition to its strong correlation with depression and state anxiety, shows a significant negative relationship with age (r = −0.24; *p* < 0.001), education (r = −0.18; *p* < 0.001), and income (r = −0.24; *p* < 0.001). These results suggest that older individuals, those with higher education, and higher income levels tend to experience lower levels of trait anxiety. Additionally, trait anxiety is positively correlated with skin color (r = 0.19; *p* < 0.001), indicating that black/brown individuals may experience higher levels of trait anxiety.

Age is positively correlated with both education level (r = 0.25; *p* < 0.001) and income (r = 0.31; *p* < 0.001), suggesting that older individuals in this sample tend to have higher levels of education and income. Finally, education level is also positively correlated with income (r = 0.37; *p* < 0.001), reinforcing the expected relationship that higher education is associated with higher income. There is no significant relationship between skin color and income or education level. The results can be seen in Table 3.

To further investigate the differences in results between black/brown and white individuals, we conducted Student’s *t*-test (Table 4). For state anxiety, black/brown participants scored higher than white participants. Similarly, for trait anxiety, black/brown participants scored higher than white participants, reflecting elevated trait anxiety among black/brown individuals. In addition, the internalized homonegativity was marginally lower for black/brown participants compared to white participants.

The effect sizes, measured by Cohen’s d, reveal varying degrees of differences between white and black/brown participants. The effect size for internalized homonegativity was small (d = 0.26), denoting a statistically significant but likely limited practical difference. Meanwhile, state and trait anxieties each had medium effect sizes (d = 0.4 for state anxiety, 0.33 for trait anxiety), suggesting that black/brown participants have substantially higher levels of anxiety than their white counterparts. Although these effect sizes, taken by themselves, are not big, they represent differences that are meaningful in that sphere of psychological research where moderate effects are often seen as potential indicators of important differences in mental health outcomes.

Hierarchical multiple linear regression analyses were carried out to assess whether skin color remained a significant predictor of psychological distress after controlling for education and income. Skin color was entered in the first step in each model, education in the second, and income in the third step. The full results can be seen in Table 5.

Skin color was a significant predictor of depression, trait anxiety, and state anxiety although causality cannot be inferred due to the cross-sectional design; its significance remained after accounting for education and income. However, the incorporation of sociodemographic variables increases the explanatory power of the models. Lower levels of education and income were also associated with increased psychological distress, although the strength and significance of the relationships varied based on the outcome.

On the other hand, the models for predicting internalized homonegativity outcomes, both the total score and the subscales of internalized stigma and perceived social oppression, did not yield statistically significant results for any of the predictors. This suggests to us that psychological distress in this sample is influenced by both socioeconomic position and racialized experience, while internalized homonegativity does not seem to have been substantially influenced by the sociodemographic variables we examined.

## 4. Discussion

Our study aimed to assess associations between sociodemographic variables, homonegativity, psychological distress, and skin color in a sample of Brazilian gay men, and to evaluate possible differences in homonegativity and psychological distress between groups of black and white gay men. It also aimed to investigate the predictive power of skin color on homonegativity and on symptoms of depression and anxiety. Our sample consisted mainly of upper-middle-class Brazilian gay men from large Brazilian urban centers, particularly São Paulo.

In the overall analysis of the participants, we found a positive and significant correlation between total homonegativity and depression. This finding supports our initial hypothesis and is consistent with other Brazilian [5,62] and international studies [20,33,51,63,64]. The association between homonegativity and depression has been explained, at least in part, by the minority stress theory [51]. This theory postulates that LGBTQIA+ people are more vulnerable to mental health problems due to the additional stress caused by chronic exposure to homonegativity in society, including social isolation, social rejection and homophobic bullying, lack of role models, and less representation in positions of power.

In addition, gay individuals may be exposed, from childhood, to various episodes of microaggression [25], which are often not clearly perceived even by the individual subjected to it, such as the invisibilization of aspects related to homosexuality by the family. All these variables, added to the stresses of everyday life, tend to produce a pervasive and chronic impact on self-esteem and psychological overload [65]. In addition, as hypothesized by Minority Stress Theory, chronic exposure to homonegativity may contribute to the internalization of this phenomenon, hindering the formation of healthy emotional bonds, reducing self-care repertoire, aggravating mental health problems, and lowering their self-esteem and overall quality of life [32,38,62,64,66].

We also found a negative and significant correlation between total homonegativity and level of education, indicating that participants with higher levels of education tended to report lower homonegativity scores. Since higher levels of education tend to be associated with higher income indicators (this association was also found in this study), it is possible that, despite the difficulties associated with homonegativity, these participants have managed to structure themselves in terms of intellectual and work development, two key factors in shaping a person’s self-concept in Western society, associated with better self-esteem and a greater sense of self-efficacy. Furthermore, it is possible that individuals with higher educational levels are more likely to have access to information about homosexuality and to critically assess religious dogmas linking homosexuality to sin—a widespread phenomenon in Brazil’s predominantly conservative and Christian culture [5]. However, it is possible that other factors, such as openness to diversity, may influence both educational attainment and homonegativity levels.

Contrary to our hypothesis, we found no correlation between internalized homonegativity and depression and anxiety among the participants. This result contradicts previous studies [38,67,68] and may be explained by the fact that the perception of homonegativity, especially internalized homonegativity, may be difficult to assess because of defense mechanisms, such as denial and projection, and may serve to protect individuals from the cognitive dissonance related to the realization of the prejudice against themselves. On the other hand, the relatively weaker association observed between homonegativity and depressive and anxiety symptoms could potentially be attributed to the relatively young age of the participants in our study and their socioeconomic status. Previous research has indicated that being younger and having more financial resources minimizes the adverse associations between perceived homonegativity and mental health problems [64,69]. Furthermore, the lack of association between internalized homonegativity and participants’ depression and anxiety may indicate the presence of moderating variables (e.g., social support, coping strategies, religiosity) that may mitigate the negative psychological effects of internalized homonegativity, as shown in previous research [4,27,30].

In addition, to assess homonegativity, we used the Brazilian version [34] of the Internalized Homophobia Scale [56], one of the few instruments with evidence of validity for the Brazilian population, to assess homonegativity perceived in the community and internalized by gay men. Although the instrument has been used in research around the world and has relatively good indicators of internal validity, it may not adequately capture new forms of homonegativity, such as microaggressions, which may be less explicit and therefore more difficult to detect [25,70].

We also found a positive and significant correlation between total homonegativity and state anxiety, a construct related to the level of anxiety the individual feels in the present. Consistent with previous studies, this finding shows that the expectation of rejection and history of punishment to which homosexuals are often subjected in heteronormative societies increases hypervigilance and alters the neurophysiology of the limbic system, making the individual more sensitive to aversive stimuli and increasing flight and avoidance behaviors, especially in interpersonal relationships [20,41,51,71,72]. It should be noted that when homonegativity is internalized by gay men, even thoughts related to homosexuality, in its various forms of manifestation, can evoke somatic and cognitive responses of anxiety [61]. Affections experienced as negative, such as fear, shame, and guilt, increase the risk of seeking maladaptive self-regulation strategies, such as alcohol and other drug abuse and compulsive sexual behavior. This may help explain, at least in part, the high prevalence of such behavioral patterns among gay men [20,21,31].

Our analysis revealed negative and significant correlations between trait anxiety and state anxiety and age and income. This result suggests that in the sample evaluated, older individuals with higher incomes reported lower levels of homonegativity. This result may be a consequence of the development of socio-emotional skills for managing anxiety throughout life, as well as engagement in mental health treatments such as psychotherapy and the use of psychotropic medication. In addition, the increase in income among Brazilian gay men may facilitate the possibility of moving to more favorable social contexts, such as larger cities, which tend to be more progressive, or even to countries with lower rates of homonegativity. Finally, we believe that higher-income gay men can protect themselves from homonegativity through a process of conditioned acceptance of the gay individual by the community, if one has socially desirable and valued characteristics, such as beauty and prosperity. The harmful effects of this process are evident in the high prevalence rate of burnout [73] and eating disorders [74], mental health problems that affect the Western gay community disproportionately, as well as self-esteem that is excessively associated with performance, to the detriment of other characteristics and values of the individual.

We found significant differences in trait and state anxiety levels between skin colors, as well as skin color proving to be a significant predictor of symptoms of depression, trait anxiety, and state anxiety. These findings may reflect the cumulative impact of stressful situations experienced throughout life, such as the Brazilian racialized heterosexist impact, may have shaped some of the participants’ personality traits [75,76,77], contributing to high levels of psychological distress in this population. Additionally, a positive relationship was found between trait anxiety and the internal and external perception of stigma, which is in line with previous studies associating homonegativity with anxiety [36,38,63]. As for the differences in anxiety averages between the two racial groups, the results confirm the findings of Lin et al. (2023) [78], indicating that black or brown gay men have higher levels of anxiety. This may be due to their exposure to multiple minority stressors compared to white individuals [79].

Nonetheless, black participants showed less perceived homonegativity and less depression. This result may be attributable to the fact that racial identity development does not in fact play a major role in explaining mental problems among brown/black gay men in Brazil, and that perhaps resilience may be a more important mitigating factor for depression. This is aligned with Minority Strengths Theory [70], which suggests that personal and collective strengths in minority populations enable the development of intersectoral resilience. This phenomenon is related to the ability of individuals and communities to adapt, recover, and thrive in the face of adversity, recognizing the interconnected impact of multiple social identities—such as race, gender, class, sexuality, and ability—on their experiences of both vulnerability and strength [69,70]. Regarding black gay men, intersectoral resilience highlight the possibility to respond to stigma and oppression with pride in intersectional identities, perseverance, community advocacy, and social support, helping them in navigating racism and heterosexism [8].

In fact, in the context of black and brown gay communities in Brazil, the capacity of individuals to recover from adversities while maintaining their mental health is particularly salient due to historical and ongoing systemic injustices faced by these populations, including discrimination, economic disparities, and social exclusion. This dual recognition allows for a more nuanced understanding of mental health factors contributing to resilience, which often include strong family and community ties, cultural and racial identity, access to resources, and positive coping strategies, fostering a sense of belonging. Moreover, the adaptability of these communities in navigating various social changes highlights their inherent strengths, which may serve as protective factors against stressors. Furthermore, black and brown communities often display remarkable adaptative strategies, such as leveraging peer networks and community organizations to facilitate mental awareness and offer mutual support. Hence, the minority strength model can encourage a shift in perspective among mental health professionals who work with black and brown gay men, steering them away from a deficit-based view and towards one that acknowledges the resilience and resourcefulness of this community.

Our results also bring light to the role racial identity may play in shaping mental health outcomes among black and brown gay men in Brazil. A strong racial identity can lead to an increased sense of community and self-esteem, which are crucial protective factors against mental health issues through identity pride and affirmation. We believe that the interplay of racial identity and mental health is multidimensional and requires targeted approaches that acknowledge the unique experiences these men face. Protective factors must take into consideration the historical context of Brazil regarding colonial legacy, and post-colonial developments, socioeconomic factors, media representation, the global LGBTQIA+ movement and local activism, access to health care and mental health support, and violence and discrimination experiences.

Hypothesis 3 was also partially confirmed. Our study showed that skin color was a significant predictor of anxiety and depression symptoms, showing that individuals with different skin colors experience higher levels of psychological distress, confirming previous findings by [64,80,81]. However, regarding homonegativity, we found no significant association. These results do not indicate that there is a biological relationship between psychological distress and race, but rather racialization [82], derived from the environment, context, and society [83], more specifically in Brazil, which is marked by social inequality [84], invisibilization [12], and violence [16], especially against black and brown people.

These circumstances are part of a wider context of social and racial inequality in Brazil, which is one of the most unequal countries in the world [84]. Social inequality in Brazil is deeply rooted in the country’s historical structure, marked by colonization and the enslavement of black peoples, the consequences of which are still felt, particularly in issues related to skin color. Although Brazilian society has made progress in terms of recognizing rights for LGBTQIA+ populations, racial discrimination and homonegativity continue to profoundly affect the lives of many individuals, especially black gay men, who are marginalized by multiple factors, including masculinity norms that are rooted in historical racialized contexts and often conflate masculinity and heterosexuality, making it difficult for black gay men to achieve societally prescribed masculine expectations.

Internalized homonegativity is recognized as a significant stress factor for LGBTQIA+ individuals [68]. In this study, we expected to identify a positive and significant correlation between homonegativity and depressive symptoms, in line with previous research [5,20,66,67,85,86]. However, although none of the homonegativity factors showed a statistically significant correlation with the indicators of psychological distress, there was a positive relationship, albeit weak, consistent with a previous study [87].

Although it is essential to investigate the relationship between internalized homonegativity and psychological distress, Frost and Meyer (2009) [85] argue that it is even more relevant to recognize that it makes no sense to approach homonegativity only from an internal perspective, as both internal and external homonegativity emerge as phenomena derived from social stigma [35]. In this sense, to promote significant changes in the sociocultural scenario, we believe that it is essential to implement intersectional training aimed at mental health professionals, service providers, and health systems, both public and private [88]. This training should aim to diversify mental health practices and interventions to understand and prioritize the specific and current needs of sexual and racial minorities in Brazil, considering aspects of the history of these groups and the social marginalization against them, from an intersectoral perspective. Therefore, we emphasize the importance of including topics related to sexual and ethnic/racial diversity during undergraduate studies in the health area, as well as advanced training, developed or certified by the Brazilian Ministries of Education and Health, for federal employees [88,89].

We believe that two parallel strands of work are essential to mitigate homonegative and racist narratives in Brazilian society and their harmful effects. The first strand has to do with investing in the identification of difficulties and mental health care for individuals who are already chronically exposed to minority stress. This measure could be developed through the creation of support groups and legal education focused on key milestones in the struggle for civil rights acquired in recent decades, and on how to act in situations where these rights are threatened or denied. In addition, classical mental health care measures such as psychotherapy and psychiatric follow-up should be considered in specific cases. The second area of work is to promote educational programs aimed at reducing sexual and racial stigma in families, schools, health services, universities, and communities in Brazil. We believe that these permanent educational programs should include the effects of sexual and racial stigma, in individual and collective terms, promoting the recognition, appreciation, and protection of sexual and ethnic/racial diversity, through mass media such as television and the internet, linked to the federal government, legitimizing and making explicit support for this population.

In the cultural sphere, it is proposed to promote cultural events, civic groups, and platforms that include black and brown LGBTQIA+ people, with the aim of making them racially inclusive spaces through cultural integration. Such initiatives could include arts festivals that celebrate racial and sexual diversity, community support groups that focus on intersectional issues, and digital platforms that amplify the narratives and stories of these populations. In addition, it is essential that these actions are supported by partnerships with civil society organizations, educational institutions and the private sector to ensure ongoing resources and visibility. It is also important to consider the challenges of implementing these initiatives, such as resistance from conservative sectors and the difficulty of accessing resources in peripheral regions. To overcome these obstacles, it would be strategic to involve community leaders, create public policies that encourage funding for inclusive cultural projects, and use social networks to extend the reach of actions. Such actions can help to transform the sociocultural landscape by giving voice and visibility to historically marginalized groups in Brazil, a society marked by a legacy of colonization, enslavement, and contemporary forms of oppression linked to white European supremacy [88].

Importantly, while this study found significant relationships between skin color and psychological distress, the decision to group black and brown individuals together as a single category may obscure important within-group differences. Brazil has a racial reality that is characterized by a binary (i.e., black versus white) as well as a continuum of phenotypes and historical regions that shape both the form and intensity of racial discrimination. Moreover, although our sample revealed statistically significant differences in income and education based on skin color, these statistical differences may not accurately reflect the complexity and depth of structural inequities for black and brown individuals in other parts of Brazil. Future studies will need to code for these racial categories and consider other intersectional variables, such as region, class, education, and phenotypic (assumed) perception, to expand understanding of the multidimensional effects of racism.

### 4.1. Study Limitations and Future Directions

Although we believe that our objectives were met, we must acknowledge the limitations of this study. Our study was cross-sectional in nature, which makes it impossible to establish causal relationships between the variables studied. This means that in our research, we cannot establish a causal relationship between skin color, depression and anxiety symptoms, and homonegativity. Our research included a non-probabilistic sample made up of gay Brazilian men with a high level of education (40% of the participants had completed postgraduate studies) and a higher income than that of the general Brazilian population (35% of the participants had an income between 1500–2250 *EUR*). This sociodemographic profile was found in both the white and black/brown gay groups and does not represent the Brazilian reality, where the majority of the population, especially black and brown people, have a low income and a low level of education, a structural social inequality that originated during European colonization and the late abolition of slavery of black and brown people that is still present in the Brazilian reality [12,88].

In addition, a possible selection bias needs to be addressed. This research was publicized on the social networks of LGBTQIA+ communities, so both digitally excluded individuals and those who do not publicly disclose their sexual orientation were underrepresented. We believe, based on clinical experience and previous research, that the prevalence of signs and symptoms of depression, anxiety, and homonegativity may be even higher in these cases. Furthermore, most of the participants in our study lived in São Paulo, the most populous, multicultural, and ethnically and racially diverse city in Brazil, where black and brown gay men are more likely to be represented, and therefore less likely to suffer from racism and homonegativity, compared to other less heterogeneous regions of the country, such as the South, which is predominantly white. We believe that sexual stigma and racism are more openly discussed phenomena in São Paulo than in less developed regions of the country, such as the North and Northeast. Thus, it is possible that black and brown gay men living in less developed regions of the country, as well as in small urban or rural areas, may face a greater burden of sexual stigma and racism. In addition, it is essential to note that living with homonegativity in rural areas may be quite different—not only is it more silenced in social invisibility and representation, but possibly even more intense and violent, it is thought, with the more powerful presence of conservative cultural and religious beliefs, combined with reduced access to mental health services and LGBTQIA+-affirmative resources. Future studies ought to investigate geographical differences, since the rural context could pose different paths of identity development, stigma internalization, and psychological distress.

Another limitation of this study is the lack of variables that could partially explain the differences found, such as discrimination and social support. Experiences with racism and access to support networks may be important in explaining the differences in scores between black/brown and white gay men. Future studies should take these variables into account for a better understanding of the mechanisms affecting the mental health of black and brown gay men. In addition, although the Internalized Homophobia Scale has Brazilian validation and relatively good internal consistency, these psychometric properties have yet to be poorly explored across racial categories; this may potentially limit our study, in that the instrument might not fully capture the extent of internalized homonegativity by skin color. Future research ought to investigate the measurement invariance of the Internalized Homophobia Scale to ensure applicability in racially and ethnically diverse populations.

Moreover, our binary classification of participants’ skin color as “white” and “black/brown”. Although we relied on IBGE categories and their justification to collect statistics, this classification may have masked significant differences within each category of skin tone. Racial identity in Brazil is shaped by many variables, such as skin tone, phenotypic characteristics, and region. Moreover, experiences of racism can differ widely in both the “black” and “brown” racial categories. Self-identification in terms of skin color is important; however, we did not document how others perceived the participants, since experiences of discrimination are often shaped by how others perceive an individual, particularly given the role of colorism in Brazil. Future studies should include more sophisticated measures of race and skin color dimensions that include both self-identified and other-classified skin color or race regarding experiences of psychosocial outcomes. Finally, we used self-report instruments to assess the variables of interest. We believe that homonegativity, especially internalized homonegativity, may be more difficult to perceive because defense mechanisms, such as denial, may serve to protect individuals from the cognitive dissonance associated with prejudice against themselves. Similarly, symptoms of depression and anxiety were also assessed by self-report and may have been underestimated by participants’ ability to perceive themselves.

These limitations suggest caution in generalizing our findings. Therefore, conducting replication studies with larger and more probable samples from all regions of Brazil, as well as longitudinal research, will be crucial to deepen our understanding of the relationship between skin color, homonegativity, and mental health among gay men in Brazil.

### 4.2. Practical Implications for Clinicians and Policymakers

Drawing from our findings, we propose the following practical implications for mental health practitioners, educators, and policymakers:

1. Train healthcare practitioners: Mental health practitioners should have ongoing training to understand sexual and racial stigma and needs in relation to gay men of color who may face unique experiences related to compounded minority stress.
2. Include LGBTQIA+ and racial diversity content in health education curricula: Colleges and technical training programs should include LGBTQIA+ and other racial and ethnic identities in foundational health education curricula to create inclusive and affirming practice.
3. Create community-based support programs: Local government and non-government organizations (NGOs) should develop support networks and community centers for LGBTQIA+ individuals, especially those living in areas lacking access to mental health services. Make access to relevant services equitable: Policymakers should make provisions to enhance equitable access to, as well as quality of, mental health services in underserved communities, especially in rural communities or periphery urban locations where stigma may be more pronounced or hidden.
4. Create public policies related to stigma and discrimination: Policy makers should create and enforce anti-discrimination policies related to the intersections of sexual orientation and race, while also providing support for affirmative policies and educational campaigns.
5. Enable access to appropriate services equitably: Policymakers should take action to increase both the equity of access to and quality of mental health services in communities that are underserved, especially in rural communities or peripheral urban locations where stigma might be more salient or covert.
6. Enable public policy related to stigma and discrimination: Policymakers should develop and enforce anti-discrimination policies that intersect sexual orientation and race while also supporting the endorsement of affirmative policies and educational programs.

## 5. Conclusions

The Brazilian gay men assessed reported high levels of community and internalized homonegativity and reported a high frequency of anxiety and depression symptoms. We found lower levels of internalized homonegativity in black and brown gay men. These findings may indicate the presence of protective mechanisms against the internalization of homonegativity and depression, but not against anxiety, which was significantly higher in this group doubly exposed to the burden of stigma. Our results reiterate the importance of developing specific psychotherapeutic interventions and intersectional educational programs, as well as promoting inclusive cultural spaces and visibility for these communities, considering the specificities of the Brazilian sociocultural reality, with the aim of reducing homonegativity and racism and its effects. These initiatives could create a more favorable social environment for the construction of positive gay identities, regardless of skin color.

## Figures and Tables

**Table 1:** Sociodemographic and clinical characterization of participants (n = 229).

		Total (n = 229)		White (n = 151)		Black/Brown (n = 78)		χ^2^	*p*	Cramer’s V
		N	%	N	%	N	%			
Education level	Complete elementary school	1	0.4	1	0.7			1.678	0.29	-
	Incomplete high school	1	0.4			1	1.3			
	Complete high school	11	4.8	5	3.3	6	7.7			
	Technical course	4	1.7			3	3.8			
	Complete higher education	27	11.8	47	31.1	20	14.1			
	Incomplete higher education	67	29.3	16	10.6	11	25.6			
	Incomplete postgraduate studies	13	5.7	7	4.6	6	7.7			
	Complete postgraduate studies	54	23.6	38	25.2	16	20.5			
	Master’s degree	24	10.5	16	10.6	7	10.3			
	Doctorate	27	11.8	20	13.2	8	9			
Employment status	Employed	200	87.3	136	90.1	64	82.1	8.611	0.03 **	0.19
	Unemployed	23	10	14	9.3	9	11.5			
	On sick leave	4	1.7			4	5.1			
	Retired	2	0.9	1	0.7	1	1.3			
Housing condition	Own	98	42.8	66	43.7	32	41	1.082	0.58	-
	Rented	118	51.5	75	49.7	43	55.1			
	Borrowed/provided	13	5.7	10	6.6	3	3.8			
Living arrangements	With family/partner	125	54.6	86	57	39	50	1.786	0.40	-
	With colleagues/friends	27	11.8	15	9.9	12	15.4			
	Alone	77	33.6	50	33.1	27	34.6			
Positive for HIV	Yes	36	15.7	23	15.4	13	16.7	0.080	0.77	-
	No	193	84.3	128	84.8	65	83.3			
Completion of the last HIV test	<6 months	117	51.3	81	53.6	36	46.8	0.969	0.32	-
	>6 months	111	48.7	70	46.4	41	53.2			
Socioeconomic status (monthly income) *	Up to BRL 4618 (EUR 750; USD 814)	92	42.2	50	35.2	42	55.3	12.124	0.03 **	0.23
	Up to BRL 9238 (EUR 1500; USD 1629)	54	24.8	40	28.2	14	18.4			
	Up to BRL 13,858 (EUR 2250; USD 2444)	21	9.6	12	8.5	9	11.8			
	Up to BRL 18,478 (EUR 3000; USD 3259)	17	7.8	12	8.5	5	6.6			
	Up to BRL 23,097 (EUR 3750; USD 4073)	12	5.5	10	7	2	2.6			
	More than BRL 23,097 (EUR 3750; USD 4073)	22	10.1	18	12.7	4	5.3			
Marital status	Single	172	75.1	195	69.5	67	85.9	7.385	0.02 **	0.18
	Married	51	22.3	41	27.2	10	12.8			
	Divorced	6	2.6	5	3.3	1	1.3			
Religion	Practicing Catholic	16	7	10	6.6	6	7.7	21.746	0.01 **	0.30
	Non-practicing Catholic	38	16.6	26	17.2	12	15.4			
	Practicing Protestant	5	2.2	3	2	2	2.6			
	Non-practicing Protestant	18	7.9	5	3.3	13	16.7			
	Practicing Spiritist	13	5.7	6	4	7	9			
	Non-practicing Spiritist	15	6.6	12	7.9	3	3.8			
	Practicing Afro-Brazilian religious	15	6.6	10	6.6	5	6.4			
	Non-practicing Afro-Brazilian religious	11	4.8	8	5.3	3	3.8			
	Atheist or agnostic	70	30.7	55	36.4	15	19.2			
	Other religions	17	4.8	10	6.6	7	9			
	Without religion	11		6	4	5	6.4			

* In 2024, the minimum wage in Brazil is *BRL* 1412 (*EUR* 229.25; *USD* 249.02). Values converted using the exchange rate of 17 October 2024. ** *p* < 0.05.

**Table 2:** Descriptive statistics and confidence intervals for study variables.

Variables	Possible Scores	Theoretical Mean	M	SD	95%CI
Depression	0–63	31.5	14.03	9.53	12.79–15.28
Homonegativity (community)	4–16	10	11.41	1.43	11.23–11.60
Homonegativity (internalized)	15–60	37.5	38.21	3.36	37.78–38.65
Homonegativity (total)	19–76	47.5	49.63	3.83	49.13–50.13
State anxiety	20–80	50.0	48.16	12.61	46.52–49.80
Trait anxiety	20–80	50.0	48.65	12.58	47.01–50.29

**Table 3:** Correlations between depression, homonegativity, anxiety, age, education level, and income.

Variables	Depression	Homonegativity (Total)	Homonegativity (Community)	Homonegativity (Internalized)	State Anxiety	Trait Anxiety	Age	Education Level	Income
Depression	-	0.13 *	0.09	0.11	0.75 **	0.80 **	−0.11	−0.19 **	−0.17 **
Homonegativity (total)		-	0.49 **	0.92 **	0.06 *	0.11	0.04	0.08	0.10
Homonegativity (community)			-	0.13 *	0.08	0.13 *	−0.06	−0.05	0.03
Homonegativity (internalized)				-	0.03	0.07	0.07	0.11	0.10
State anxiety					-	0.87 *	−0.15 *	−0.11	−0.16 *
Trait anxiety						-	−0.24 **	−0.18 **	−0.24 **
Age (years)							-	0.25 **	0.31 **
Education level								-	0.37 **
Income									-

** *p* < 0.001, * *p* < 0.05.

**Table 4:** Differences between groups by skin color for depression, homonegativity, and anxiety.

Variable	Total (n = 229)		White (n = 151)		Black/Brown (n = 78)				
	M	SD	M	SD	M	SD	t	*p*	d
Depression	14.03	9.53	13.36	9.66	15.34	9.21	1.494	0.13	-
Homonegativity (community)	11.41	1.43	11.37	1.48	11.50	1.34	0.610	0.54	-
Homonegativity (internalized)	38.21	3.36	38.52	3.03	37.62	3.87	−1.920	0.05 *	0.26
Homonegativity (total)	49.63	3.83	49.90	3.52	49.12	4.35	−1778	0.07	-
State anxiety	48.16	12.61	46.46	12.69	51.46	11.85	2.893	0.004 *	0.40
Trait anxiety	48.65	12.58	47.25	12.62	51.36	12.12	2.365	0.001 *	0.33

* *p* = <0.05.

**Table 5:** Multiple hierarchical linear regression.

Dependent Variable	Model	Predictor	β	*p*	R^2^ adj.
Depression	1	Skin color	0.170	**0.010**	0.025
	2	Skin color	0.176	**0.007**	0.059
		Education	−0.197	**0.002**	
	3	Skin color	0.179	**0.006**	0.068
		Education	−0.152	**0.028**	
		Income	−0.119	0.086	
Trait anxiety	1	Skin color	0.190	**0.004**	0.032
	2	Skin color	0.196	**0.003**	0.064
		Education	−0.190	**0.003**	
	3	Skin color	0.201	**0.002**	0.097
		Education	−0.114	0.096	
		Income	−0.206	**0.003**	
State anxiety	1	Skin color	0.146	**0.027**	0.017
	2	Skin color	0.150	**0.023**	0.027
		Education	−0.120	0.068	
	3	Skin color	0.153	**0.019**	0.042
		Education	−0.065	0.356	
		Income	−0.148	**0.035**	

Note: Standardized beta coefficients (β) are shown. Each model adds one predictor at a time: Model 1 includes skin color; Model 2 adds education; Model 3 adds income. Bold values indicate statistically significant predictors (*p* < 0.05).

## Data Availability

Data will be made available upon request.
